# Anti-inflammatory and in-vitro antibacterial activities of Traditional Chinese Medicine Formula Qingdaisan

**DOI:** 10.1186/s12906-016-1475-4

**Published:** 2016-12-05

**Authors:** Xinghua Zhao, Xin He, Xiuhui Zhong

**Affiliations:** 1 College of Traditional Chinese Veterinary Medicine, Agricultural University of Hebei, No. 2596 Lekai South Street, Baoding, Hebei 071000 People’s Republic of China; 2 Institute of Traditional Chinese Veterinary Medicine, Agricultural University of Hebei, No. 2596 Lekai South Street, Baoding, Hebei 071000 People’s Republic of China

**Keywords:** Qingdaisan, Anti-inflammatory activity, Dental ulcer, In vitro antimicrobial effect

## Abstract

**Background:**

Qingdaisan (Formulated Indigo powder, QDS) are widely used for treatment of aphtha, sore throat and bleeding gums in China. The aim of the study is to evaluate the anti-inflammatory, antibacterial and dental ulcer therapeutic effects of QDS.

**Methods:**

Dimethylbenzene-induced ear edema test and cotton pellet-induced granuloma test were used to evaluate anti-inflammatory activities of QDS on acute and chronic inflammatory. The healing time and local pathologic changes were used to assess the therapeutic effects of QDS on dental ulcer. The antibacterial activities of each component and the whole formulation of QDS were determined by agar well diffusion assay. High-dose and low-dose QDS were tested in this experiment and Gui Lin Watermelon Frost Powder (GLWFP) was used as positive control.

**Results:**

Oral treatment with QDS significantly accelerated the healing of ulcerative lesions induced by phenol injury. The dental ulcers of high-dose QDS group were all healed within 6 days. It was shorter than those of low-dose QDS group and GLWFP group. Less quantity of inflammatory cells and plenty fibroblasts were observed in pathological section of QDS groups. QDS also exhibited significant anti-inflammatory activity both in acute and chronic animal models. Although some of the components exhibited antibacterial activities, the whole formulation of QDS didn’t show any significant antibacterial activity in vitro.

**Conclusion:**

The study showed that QDS has obviously anti-inflammatory activity for both acute and chronic inflammatory, also has a remarkable effect for healing dental ulcer caused by phenol. QDS didn’t have antibacterial activity to selected strains in vitro.

## Background

Mouth ulcer is a common inflammatory disease characterized with ulcer on the mucous membrane combined with soreness, edema, bleeding, pain and sometimes difficulties in swallowing. Many factors can be involved in mouth ulcer’s development and progression, such as microbial infection, psychological stress, and hormonal state and immunological abnormalities [1]. Mouth ulcers are also associated with many diseases and medical therapeutics [2–4]. According to the American National Cancer Institute, 40–80% of patients receiving standard dose chemotherapy will suffer form mouth ulcer as a common side effects [5]. Mouth ulcer may be recurrent in special case which is called aphthous ulcer or recurrent aphthous stomatitis, it give the patient great pain since it occurs recurrently and beacuse the etiology and pathogenesis remains unclear, there is no consensus regarding a definitive curative therapy [6]. There are many western preventive and therapeutical interventions for mouth ulcer, topic treatments are corticosteroids, antibiotics, and analgesics. But they all may cause side effects when used for longer duration and in high frequency.

Herbal medicine is widely used in Traditional Chinese Medicine (TCM) to treat mouth ulcers over thousand years with slight side effects. It may be used as single herb or herbal formula. Qingdaisan (Formulated Indigo Naturalis powder, QDS) is a traditional Chinese classical prescription, which is published in the Chinese Veterinary Pharmacopoeia 2005 [7]; it is used for stomatitis, gingivitis, open sores, sore throat, ulcerative colitis and so on [8–10]. The formula of QDS includes Indigo naturalis, *Coptis*, *Phellodendron*, *Mentha*, *Platycodon* and *Acacia* with equivalent weight. Indigo naturalis was used for exanthema and ulcers in TCM. It is effective in treating psoriasis and other skin diseases [11]. Indigo naturalis was found to inhibit superoxide anion generation, the activation of MAPK, also regulate calcium mobilization [12]. Pretreatment with indigo naturalis extract could attenuate TNF-α-induced increase in Jurkat T cell adhesion to human umbilical vein endothelial cells (HUVECs) as well as decreased the protein and messenger (m) RNA expression levels of vascular cell adhesion molecule-1 (VCAM-1) on HUVECs [13]. Coptidis rhizoma was regularly used in inflammatory and septic processes. It was found to inhibit IL-1α, IL-6, iNOS expression, and NO production in RAW 264.7 macrophages [14]. *Phellodendron* was traditionally considered as anti-toxic herb and was used for abscesses and sores. It can inhibit TNF-α, IL-1β, and iNOS production, as well as phosphorylation of extracellular-signal regulated kinases (ERK) and NF-κB activation in microglia cells [15]. *Acacia catechu* is clinically used for tissue regeneration, wound healing, sores and abscesses as well as mouthwash fororal ulcers [16]. *Platycodon* can reduce the iNOS and COX-2 gene expression through blocking of NF-κB activation [17]. Though there is many clinical evidence of the efficacy of QDS on Mouth ulcer, the research data is very limited. In this study, we aimed to investigate the anti-inflammatory effects of QDS.

## Methods

### Plant material and bacteria

Indigo Naturalis, extraction of leaf of *Isatis indigotica* Fortune (*Qing Dai*), *Coptis*, root of *Coptis chinensis* Franch (*Huang Lian*), *Phellodendron*, bark of *Phellodendron chinense* C.K.Schneid (*Huang Bai*), *Mentha*, leaf and stem of *Mentha haplocalyx Briq.* (*Bo He*), *Platycodon*, leaf and stem of *Platycodon grandiflorus* (Jacq.) A.DC (*Jie Geng*), and *Acacia,* extraction of peeled trunk and branch of *Acacia catechu* (Linn.f.) Willd (*Er Cha*) were all purchased from medicine market in Anguo, Baoding, China. The whole plant materials of the six herbal materials were also obtained from their original source and botanic identification was confirmed by Professor Zhenying Fu (Agricultural University of Hebei, Baoding, China). The voucher specimens (SF1-6) were deposited at Traditional Chinese Medicine specimens’ room at College of Veterinary Medicine of Agricultural University of Hebei.

According to the methods of Chinese Veterinary Pharmacopoeia 2005, the identification of Indigo Naturalis (*Qing Dai*), *Coptis* (*Huang Lian*), *Phellodendron* (*Huang Bai*), *Mentha* (*Bo He*), *Platycodon* (*Jie Geng*), and *Acacia* (*Er Cha*) were carried out by Thin Layer Chromatography (TLC) method.

The formula of QDS includes *Coptis*, *Phellodendron*, *Mentha*, *Platycodon* and *Acacia*, with equivalent weight (100 g); they were smashed into superfine powder and passed through 120 mesh screen. Then Indigo Naturalis was added into the powder with the same weight. The prepared QDS was stored at room temperature, and QDS suspension was prepared by suspending QDS in normal saline (NS).

Gui Lin Watermelon Frost Powder (derived from processed product of watermelon and glauber salts GLWFP) was purchased from Guilin Sanjin Pharmaceutical Co., Ltd, batch number 110402. The contents of Gui Lin Watermelon Frost Powder are *Mirabilitum Praeparatum, Phellodendri Cortex, Borax, Coptidis Rhizoma, Bulbus Fritillariae Thunbergii,* Indigo Naturalis*, Glycyrrhizae Radix, Radix* et *Rhizoma Rhei, Scutellariae Radix, Borneolum, Belamcandae Rhizoma, Fructus Sapindi Mukorossi, Sophorae, Tonkinensis Radix* and Menthol*.* The final formulation was made by adding porphyrized powder of *Mirabilitum Praeparatum, Borax,* Indigo Naturalis, and Menthol into fine powder of other herbal materials. GLWFP was used as a positive control drug, commercially available and documented in the Chinese Veterinary Pharmacopoeia 2005. Acetylsalicylic acid (ASA) purchased from Sigma Chemicals (St. Louis, MO) and dissolved it was NS.

The bacterial strains supplied by microbiological lab of Agricultural University of Hebei were used: *Shigella, Staphylococcus aureus, Escherichia coli, Salmonella,* and *Staphylococcus.*

### Animals

Experiments were conducted using adult male Kunming mouse (18~22 g) and young rabbits, housed in HEPA-filtered air and a constant climate (room temperature 21 ± 2 °C and relative humidity 40~70%) with a 12 h light/dark cycle (light on at 8:00 am). The animals were acclimatized to the laboratory for 1 week before testing and were used only once throughout the experiments. The animal care and treatment were conducted according to the “Principles of Laboratory Animal Care” (NIH publication #85-23, revised in 1985); the protocols were approved by the Animal Welfare Committee of Agricultural University of Hebei.

### Anti-inflammatory assays

#### Xylene-induced ear edema test

The Xylene-induced ear edema tests were used to determine the anti-inflammatory activity of QDS against acute inflammation [18]. On the first day, the mice were divided into four groups (*n* = 6), received one of the following treatments i.g.: NS, ASA, QDS low dose (300 mg/kg BW in NS suspension) and QDS high dose (600 mg/kg BW in NS suspension). Mice were given the drugs for 5 consecutive days once a day. On the sixth day, xylene (0.02 mL per mice) was smeared on the right ear. After 1 h, the mice were euthanized by cervical dislocation. A 6-mm (diameter) hole punch was used to punch out discs from both the treated as well as the control ears. The two punches were weighed immediately by analytical balance (Shanghai Minqiao Precise Science Instrument Co., Ltd, Shanghai, China), and the difference in weight was used to asses the inflammatory response. The ear swelling was expressed as the volume difference between the xylene treated ear (right ear, *Vf*) and non-treated ear (left ear. *V0*). The percentage inhibition of ear swelling was expressed as difference between drugs and NS treated Mice. The formula was showed as following:

$$ percentage\  inhibition = \frac{\left(Vf-Vo\right) control-\left(Vf-Vo\right) experimental}{\left(Vf-Vo\right) control} $$

#### Cotton pellet-induced granuloma test

Cotton pellet-induced granuloma test was used to determine the anti-inflammatory activity of QDS against chronic inflammation [19]. For this test, a sterilized cotton pellet (10 ± 1 mg) was subcutaneously introduced in the dorsum of mice anaesthetized with avertin (1 mL/kg, i.p.). After dehairing, the animals were divided into four groups (*n* = 6 in each group). The animals were treated by gavage of NS, ASA and QDS suspension (low dose and high dose) once a day for 7 consecutive days. On day 8, we sacrificed the animals, dissected the pellets out, and weighed them to obtain the wet weight. We dried the wet pellets at 60 °C overnight to determine the final dry weight. The difference between the initial (10 mg) and final dry weight (T) was considered to be the weight of the granulomatous tissues produced. We calculated the level of inhibition of granuloma tissue development using the following formula [20].

$$ inhibition\  ratio=\frac{Tcontrol- Texperimental}{Tcontrol} $$

### Dental ulcer model test

Dental ulcer model test was used to determine the treatment effect of QDS against dental ulcer. Dental ucler models were induced by 90% phenol on oral mucosa of healthy rabbits. After treatment, the buccal mucosa appearing swollen, red or visible ulcer indicated successful models. The model animals were divided into four groups (*n* = 6). The ulcer area were coated with smear of NS, GLWFP (120 mg) and QDS low-dose (60 mg) QDS high-dose (120 mg) for 5 consecutive days, low-dose QDS were applied twice a day, each time with 30 mg, GLWFP and high-dose QDS four times a day, each time with 30 mg. On 1 d, 2 d, 3 d, 4 d, 6 d, 7 d and 9 d, the diameter of the dental ulcer was measured and was used to calculate the area. Local symptom and the degree of blood engorgement of the dental ulcer were measured by a method proposed by Xiao [21]. On the sixth day, half animals of each group were sacrificed and the skin tissues at the ulcer area were excised. Histological changes of the tissues were observed through HE stain.

### Antibacterial activity test

Water extracts of each herb and QDS were obtained by adding 20 g powdered materials in 500 mL distilled water, mixing and boiling for 30 min. It was then filtered through 8 layers of muslin cloths and centrifuged at 500 g for 10 min. The supernatant was collected, this procedure was repeated twice. The supernatant collected was pooled together and concentrated to make the final concentration equivalently 1 g raw herb per milliliter. The extract was stored at 4 °C until use.

The strains were grown and maintained on nutrient agar and nutrient broth. Agar well-diffusion assay was used to test antibacterial activities of water extracts as described by Francis [22].

### Statistical analysis

The data were processed with one-way analysis of variance (ANOVA), followed by Dunnett’s multiple comparison tests. The results were expressed as mean ± SD and differences were considered significant when *p* < 0.05.

## Results

### Qualitative determination of QDS decoction

**Fig. 1:**
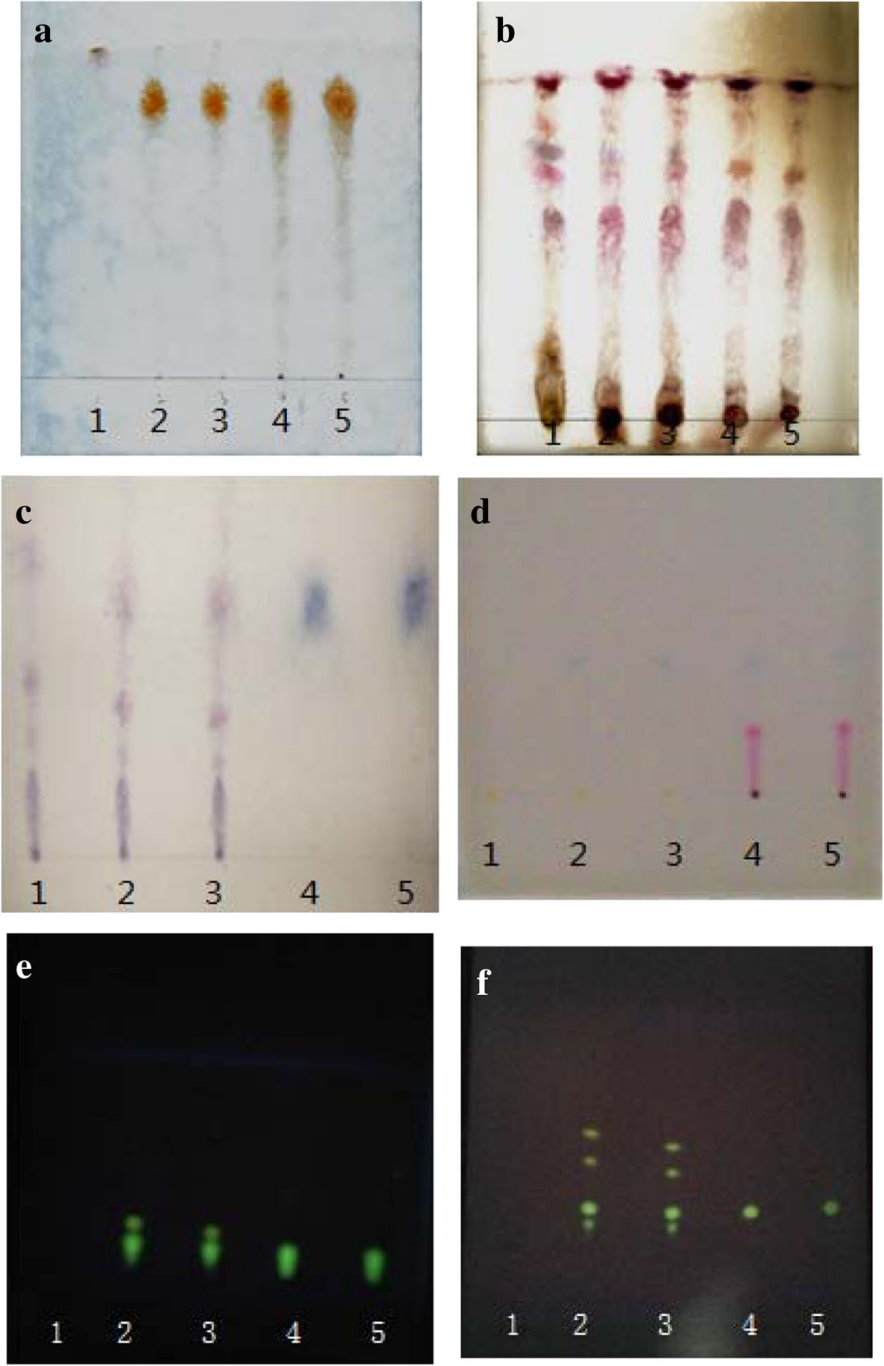
Thin-layer chromatogram of *Acacia, Platycodon, Mentha,* Indigo Naturalis*, Coptis, Phellodendron.* Note: **a**: *Acacia*, **b**: *Platycodon*, **c**: *Mentha*, **d**: Indigo Naturalis, **e**: *Coptis*, **f**: *Phellodendron*, No. *1* is negative control, *2*, *3* are test herbs, *4*, *5* are standard herbs

In chromatogram of sample solution, the same color spots showed in corresponding position with standard herb solution. In chromatogram of negative solution, there was no spot found in corresponding position (Fig. 1).

### Dimethylbenzene-induced ear edema test

The results of the dimethylbenzene-induced ear edema test were presented in Table 1. The ASA group reduced the ear edema by 34.63% compared with the negative control group; while QDS low group and QDS high group reduced edema by 31.86 and 50.38% respectively (Table 1). The swelling degree of QDS high-dose group was extremely lower than those of other groups (*p* < 0.05).

**Table 1:** Anti-Inflammatory Activity of QDS on Acute Inflammation (*n* = 6, $\overline{X}\pm SD$)

Groups	Ear quality (mg)		swelling (mg)	Percentage inhibition (%)
	left	right		
NS	8.14 ± 0.16	16.07 ± 0.62	7.94 ± 0.52	—
ASA	8.14 ± 0.13	13.33 ± 0.31*	5.19 ± 0.22*	34.63
QDS Low dose	8.11 ± 0.31	13.59 ± 0.26*	5.49 ± 0.39*	31.86
QDS High dose	8.15 ± 0.15	12.09 ± 0.36*#	3.94 ± 0.35*#	50.38

*differs significantly (*p* < 0.05) when compared against the saline-treated group (negative control). # differs significantly (*p* < 0.05) when compared against the ASA (positive control) group

### Cotton pellet-induced granuloma test

QDS exhibited anti-inflammatory activity against granulomatous edema when assessed using a chronic model of inflammation (Table 2). Interestingly, QDS suspension exhibited a nondose- dependent inhibition of granuloma formation when compared with control group. There was no significant difference between the high-dose and low-dose QDS group; the inhibition of granuloma formation was 29.57 and 36.96%, respectively. But the effect of QDS was better than the ASA.

**Table 2:** Anti-Inflammatory Activity of QDS suspension on Chronic Inflammation (*n* = 6, $\overline{X}\pm SD$)

Groups	Mean difference of cotton pellet weight (mg)	% Inhibition
NS	7.17 ± 0.39	—
ASA	5.18 ± 0.99*	27.75
QDS Low dose	5.05 ± 0.77*	29.57
QDS High dose	4.52 ± 0.48*	36.96

*differs significantly (*p < 0.05*) when compared against the saline-treated group (negative control)

### Dental ulcer model test

#### Curative effect observation

At 24 h after modeling, ulcer was formed at the cauterized area in buccal mucosa of rabbits. The ulcer area was round, neat edge and pitting defect. The surface was red and covered with yellow film. The diameter was about 7.0 mm. In ulcer periphery tissue, hyperemia, and edema were seen. The day after treatment, there was improvement in the high dose QDS group, but there was no obvious change in other groups. On the third day post treatment, each group had huge improvement in areas except the model group. For example, the area of high dose QDS group became small; the average diameter was 2.9 mm. The yellow film and swelling of mucosal was reduced. Meanwhile the hyperemia was relieved.

**Fig. 2:**
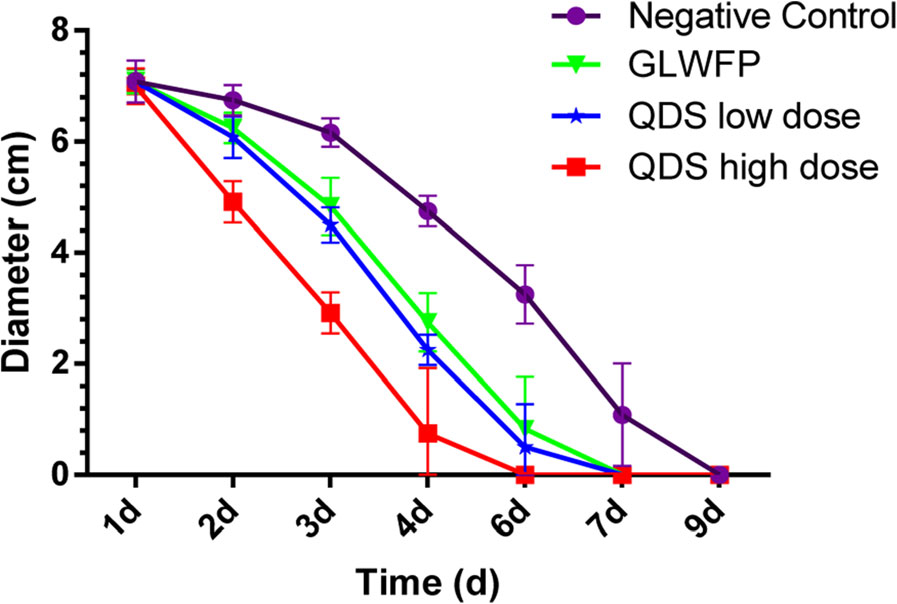
Treatment effect of QDS on phenol-induced dental ulcer in rabbits. Dental ulcer was induced by phenol and then was coated with smear of NS, GLWFP and QDS twice a day but the high-dose QDS group animals were treated with four times of QDS a day for 5 consecutive days

On day 4, four rabbits were healed and the average diameter of the other two was 2.3 mm in QDS high dose group. The average diameter was 2.8 mm in GLWFP group; the yellow film, the tissue swelling and the hyperemia were reduced compared with negative control group. On the sixth day after treatment, all the rabbits of QDS high dose group were healed. Four rabbits healed in the low dose and three rabbits healed in GLWFP group. All of the last rabbits were healed on day 9 (Fig. 2).

#### Pathological observation

**Fig. 3:**
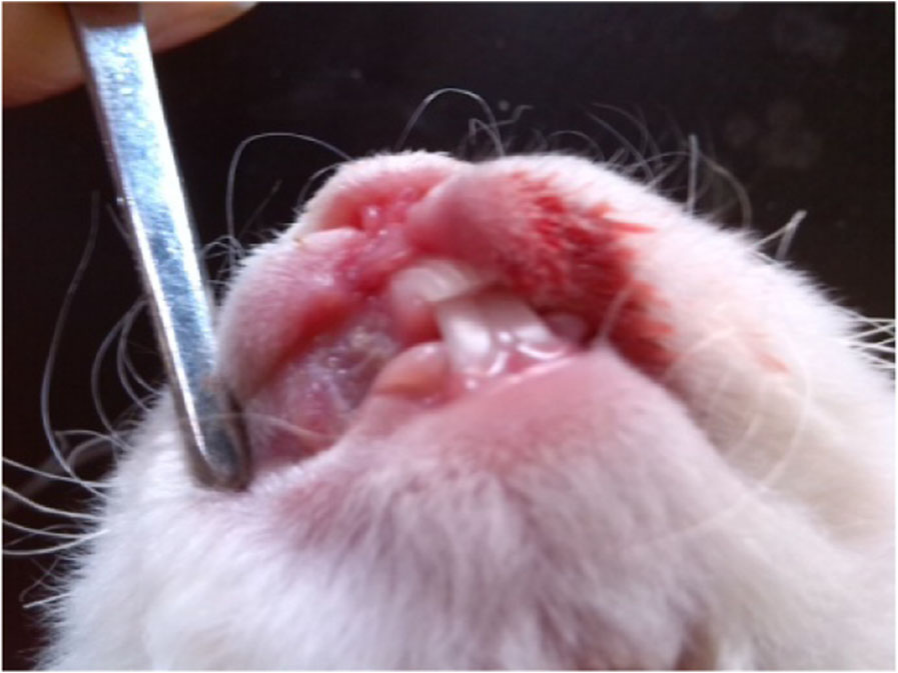
Copy rabbits dental ulcer model by phenol

**Fig. 4:**
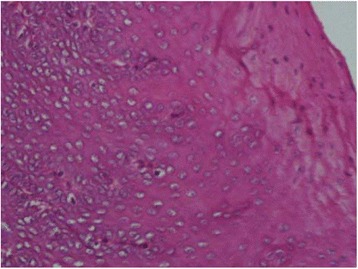
HE staining of oral mucosal in blank control group. The integrity of mucosa epithelial which lined by squamous epithelium was keep well. Under the mucous membrane was the loose connective tissue (HE×200)

**Fig. 5:**
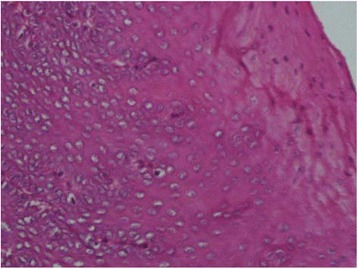
HE staining of oral mucosal in negative control group. The oral mucous epithelial cell to abscise and a number of infiltrating inflammatory cells such as lymphocytes and neutrophils could be observed (HE×200)

**Fig. 6:**
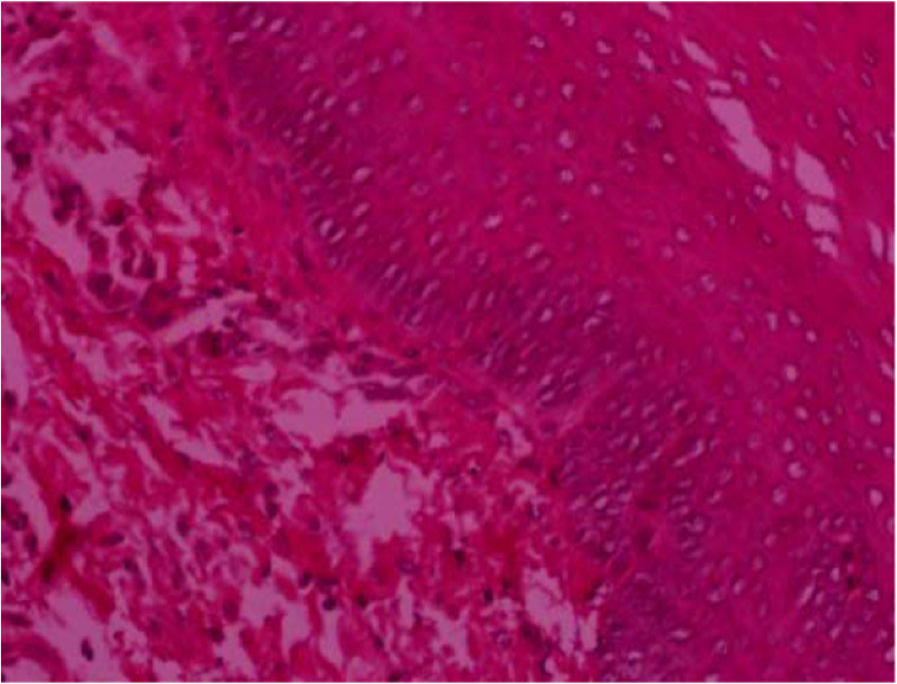
HE staining of oral mucosal after cured using QDS, Squamous epithelial integrity and thickening (HE×200)

Dental ulcers were copied in healthy rabbits by phenol (Fig. 3). The mucosa histopathology of rabbits in each group was examined. In the blank control group (Fig. 4), the integrity of mucosa epithelial kept well. The mucous membrane was lined by squamous epithelium. Under the mucous membrane was the loose connective tissue. In the model control group, ulcer caused the oral mucous epithelial cell to abscise and affect the integrity of mucous epithelium (Fig. 5). A number of infiltrating inflammatory cells such as lymphocytes and neutrophils could be observed. The day after treatment, in the high dose QDS group (Fig. 6), a small quantity of chronic inflammatory cells had infiltration in the lamina propria and group plenty fibroblasts were observed. The second day after treatment, in the GLWFP group, a small quantity of inflammatory cells had infiltration in the lamina propria and a few fibroblasts were observed. The fourth day after treatment, in the high dose QDS group, most of ulcers healed. Squamous epithelial cells were maintained normal. Under the mucous membrane was the loose connective tissue. On the sixth day after treatment, the pathohistological examination showed that infiltration of inflammatory cells was significantly reduced, plenty of fibroblasts were seen and the formation of granulation tissue also occur in the low dose QDS group.

### Antibacterial activity test

The six herbs of QDS components and QDS were tested for their antibacterial activity. The results of diameter of antibacterial circles were summarized in Table 3. The most active herb was *Coptis*, it exhibited best activity against all the tested strains except *Salmonella. Phellodendron* and *Acacia* exhibited mild antibacterial activities on two bacterial species. But interestingly, QDS didn’t show any antibacterial activities to all the strains tested. That may be because the concentration of active components in the whole formula is lower than in the single herb.

**Table 3:** Diameter of antibacterial circles of each herb and QDS

Group	Diameter of antibacterial circles (mm, mean ± SD)				
	*Escherichia coli*	*Staphylococcus*	*Salmonella*	*Staphylococcus aureus*	*Shigella*
*Coptis*	12 ± 1.71a	22 ± 2.15a	6 ± 0.00	19 ± 3.46a	18 ± 2.25a
*Phellodendron*	6 ± 0.00b	14 ± 1.44b	6 ± 0.00	12 ± 1.96b	6 ± 0.00b
*Acacia*	6 ± 0.00b	10 ± 2.71c	6 ± 0.00	11 ± 0.89c	6 ± 0.00b
*Mentha*	6 ± 0.00b	6 ± 0.00d	6 ± 0.00	6 ± 0.00d	6 ± 0.00b
*Platycodon* Indigo	6 ± 0.00b	6 ± 0.00d	6 ± 0.00	6 ± 0.00d	6 ± 0.00b
Naturalis	6 ± 0.00b	6 ± 0.00d	6 ± 0.00	6 ± 0.00d	6 ± 0.00b
QDS	6 ± 0.00b	6 ± 0.00d	6 ± 0.00	6 ± 0.00d	6 ± 0.00b
Negative control	6 ± 0.00b	6 ± 0.00d	6 ± 0.00	6 ± 0.00d	6 ± 0.00b

Different lowercase in the same line means significant difference (*p* < 0.05)

## Discussion

QDS is a traditional prescription to treat stomatitis in China that has been widely used for several centuries. Our study verified that QDS possesses anti-inflammatory effects in acute and chronic inflammation. Inflammation is a complex process that involves several events, such as: enzyme activation, mediator release, extravasations of fluid, cell migration, tissue breakdown, and repair [23]. This fact makes the use of different experimental models essential when conducting pharmacological trials. Xylene can cause instant irritation to the mouse’s ear which leads to fluid accumulation and triggers an acute inflammatory response. In our study, QDS suspension was found to significantly decrease the edema induced by xylene. Suppression of this response is likely an indication of antiphlogistic effect [24, 25].

Acute inflammation continues with the formation of proliferative cells and becomes chronic inflammation. The cotton pellet-induced granuloma test is a model for studying the transudative and proliferative components of chronic inflammatory processes [26]. After several days of cotton pellet implantation, giant cells and undifferentiated connective tissue can be observed in addition to the fluid infiltration histologically. The granuloma formation, occurring by means of the development of proliferative cells, is a chronic inflammatory process that arises due to the failure of the acute response to eliminate proinflammatory agents. The transudative components of chronic inflammation are due to the infiltration of neutrophils and exudation of fluid [27]. In the present study, QDS suspension reduced the weight of granulomatous pellets. Comparison of all doses of QDS activity with the ASA, which inhibited chronic inflammation by approximately 27.75%, revealed an insignificant (*p* > 0.05) difference, suggesting that QDS suspension was effective in chronic inflammatory conditions, and the effect was equal in strength to that of the ASA.

Dental ulcer models were commenly induced by acid and alkali. In this experiment, phenol (carbolic acid) was used to build the model. This model makes the local ashen white, red, and the ulcers were formed after 24 h [28]. Observation indexes mainly include the ulcer area, the symptoms and pathological changes. The model operation is simple, suitable for large-scale animal experiments, can be certain to promote healing of the oral mucosa and anti-inflammatory drugs screening model. But using carbolic acid is not suitable for the recurrent oral ulcer (ROU) models [29]. Continuous drug administration for 5 days is because of the natural healing process of ulcer which induced using this method is about 10 days [30].

Dental ulcer was called aphtha in traditional Chinese medicine (TCM) appears to be superficial ulcer in the oral mucosa, the size can be from a grain of rice to soybean volume into a round shape or ovoid, the ulcer surface was concave and congestive. Ulcer has the characteristics of periodic, recurrent and self-limited, often occurred in lip, buccal, tongue edge, etc. Ulcer might affect eating and its common combined symptoms included decrease feed intake, bad breath and constipation. Therefore, it is very important on the study of dental ulcer.

TCM holds that dental ulcer is always associated with the generally visceral dysfunction though it is always occurred with local [31]. Heat accumulated in the interior flares up and burns the local tissues leading to open sores, ulceration, stomatitis, swelling, and pain. According to TCM theory, Indigo Naturalis functions as the king herb to clear heat, detoxify, and resolve stagnation to relieve pain. Serving as the minister herbs, *Coptis* and *Phellodendron* clear heat and detoxify. *Acacia* and *Mentha* clear heat to relieve sore throat, astringe wounds to promote healing, serving as the adjuvant herbs. *Platycodon* which considered being the transporter to the upper jiao functions as the messenger herb and relieves sore throat. As a group, these herbs are antipyretic, detoxifying, and can reduce swelling and produce analgesia.

QDS could quicken the healing speed of dental ulcer caused by phenol and shorten the clinic healing time. QDS could improve infection status of ulcer surface especially in the early stages.

## Conclusion

The present study provided the evidence for the in vivo anti-inflammatory activity of QDS in acute and chronic inflammation. Further studies are needed to clarify the mechanisms responsible for the anti-inflammatory activity of QDS; and will focus on the effects of QDS on inflammatory factor in vivo or on RAW 264.7 cell treated with lipopolysaccharide (LPS). The results also support the claims of traditional Chinese medicine practitioners about the use of QDS in inflammatory diseases.

## Abbreviations

ANOVA: One-way analysis of variance
ASA: Acetylsalicylic acid
BW: Body weight
COX-2: Cyclooxygenase (cox)-2
ERK: Extracellular-signal regulated kinases
GLWFP: Gui lin watermelon frost powder
HUVEC: Human umbilical vein endothelial cell
IL-1: Interleukin-1
IL-6: Interleukin-6
iNOS: Inducible nitric oxide synthase
LPS: Lipopolysaccharide
NF-κB: Nuclear factor kappa B
NIH: National Institutes of Health
NO: Nitric oxide
NS: Normal saline
QDS: Qingdaisan (Formulated Indigo powder)
RAW 264.7: RAW 264.7 macrophages
ROU: Recurrent oral ulcer
TCM: Traditional Chinese Medicine
TNF-α: Tumor necrosis factor
VCAM-1: Vascular cell adhesion molecule-1
